# An Engineered Plant Metabolic Pathway Results in High Yields of Hydroxytyrosol Due to a Modified Whole-Cell Biocatalysis in Bioreactor

**DOI:** 10.3390/metabo13111126

**Published:** 2023-11-03

**Authors:** Glykeria Mermigka, Aikaterini I. Vavouraki, Chrysoula Nikolaou, Ioanna Cheiladaki, Michail Vourexakis, Dimitrios Goumas, Filippos Ververidis, Emmanouil Trantas

**Affiliations:** 1 Laboratory of Biological and Biotechnological Applications (LBBA), Department of Agriculture, School of Agricultural Sciences, Hellenic Mediterranean University (HMU), GR71410 Heraklion, Greece; gmermigka@hmu.gr (G.M.); avavouraki@hmu.gr (A.I.V.); xrica.nikolaou@gmail.com (C.N.); icheiladaki@hmu.gr (I.C.); gf6986@edu.hmu.gr (M.V.); dgoumas@hmu.gr (D.G.); 2 Agri-Food and Life Sciences Institute (Agro-Health), HMU Research and Innovation Center, GR71410 Heraklion, Greece

**Keywords:** metabolic engineering, hydroxytyrosol, bioreactor, microbial biotechnology, synthetic biology, tyrosinase, aromatic acetaldehyde synthase, aldehyde reductase, HPLC-MS/MS, whole cell biocatalysis

## Abstract

Hydroxytyrosol (HT) is a phenolic substance primarily present in olive leaves and olive oil. Numerous studies have shown its advantages for human health, making HT a potentially active natural component with significant added value. Determining strategies for its low-cost manufacturing by metabolic engineering in microbial factories is hence still of interest. The objective of our study was to assess and improve HT production in a one-liter bioreactor utilizing genetically modified *Escherichia coli* strains that had previously undergone fed-batch testing. Firstly, we compared the induction temperatures in small-scale whole-cell biocatalysis studies and then examined the optimal temperature in a large volume bioreactor. By lowering the induction temperature, we were able to double the yield of HT produced thereby, reaching 82% when utilizing tyrosine or L-DOPA as substrates. Hence, without the need to further modify our original strains, we were able to increase the HT yield.

## 1. Introduction

Hydroxytyrosol (3,4-dihydroxyphenylethanol, HT) is a monophenolic bioactive compound that belongs to the class of phenylethanoids, which are naturally occurring plant compounds with antioxidant properties [1,2]. HT is found in olives [3], olive oil [4] as well as in olive mill waste waters [5] and has been credited with a long list of beneficial effects on human health. On top of the list is its strong antioxidant activity, even compared to other popular antioxidants such as ascorbic acid or coenzyme Q10. More interestingly, HT has high antioxidant activity and is rapidly absorbed into the bloodstream [6]. Additionally, it has been documented to bestow additional biological effects, such as its ability to lower blood pressure, reduce inflammation and lower the risk of cancer and cardiovascular disease [7]. According to the European Food and Safety Administration, HT protects from oxidative damage when consumed daily (EFSA Panel on Dietetic Products, Nutrition and Allergies (NDA)) [8]. Moreover, it has been claimed that HT possesses antibacterial and antiviral activities [9,10,11], albeit there are conflicting results in this area [12]. All the above-mentioned properties declare HT a promising natural active compound of high additive value for commercial use.

Various methods have been developed to either extract HT from natural sources such as olives, olive leaves and olive mill waste waters or through de novo production. The extraction from natural sources approach possesses a number of disadvantages, including high extraction costs due to low yields, low purity due to rich sample matrices, or unstable availability due to climate and seasonal fluctuations [13]. De novo production entails chemical synthesis or bioconversion from selected precursor molecules. Related to the bioconversion process of HT production, various bacteria and fungi have been used for the biochemical conversion of precursor compounds into HT (reviewed in [14]). 

An alternative way to achieve commercial optimization of HT biosynthesis is to reconstruct the metabolic pathway within genetically engineered microbial factories [13]. The utilization of microorganisms for biological synthesis offers the advantage of eliminating the need for costly cofactors or the need to select for regioselectivity or stereoselectivity, since most enzymes provide regioselectivity and stereoselectivity [15]. In all these cases, the precursor molecules used were either simple carbon sources, like glucose [16,17,18], or substrates with structural similarity to HT such as L-tyrosine [15,17,18,19] or tyrosol [20,21,22]. In most of these reports, the biocatalysis process was conducted on a small scale.

The aforementioned strategies in metabolic engineering, together with the significant commercial value of HT, make the utilization of biotechnological methods attractive, especially because biotechnological methodologies have the capacity to provide an exceptional purity of the final products. This is an advantage compared to conventional chemical synthesis, since it eliminates the need for heavy metal catalysts or the utilization of harsh reaction conditions. A bioconversion process that involves the use of precursor compounds exhibiting structural similarities to HT has been also documented to achieve high volumetric productivity and product titer in a single step. However, the cost of the substrate poses a significant obstacle to scaling up industrial production using such methods [14].

In a previous work, we established the production of HT by *Escherichia coli* by implementing an artificial biosynthetic pathway taking advantage of both the monophenol and diphenol route [17] of tyrosine bioconversion. The dual specificity of the utilized tyrosinase (TYR) and aromatic acetaldehyde synthase (AAS) along with the utilization of an *E. coli* native aldehyde reductase created a dual metabolic pathway starting from tyrosine. A collection of HT-producing *E. coli* strains from various substrates (glucose, tyrosine, and L-DOPA) were constructed and evaluated in flask experiments. The result was the efficient redirection of metabolic flow from glucose into HT at up to 271 mg/L.

To date, there are only a few reports that use bioreactors for HT bioconversion [22,23,24,25], which is the ultimate goal in terms of the commercial exploitation of biotechnologically produced bioactive compounds. The aim of the present study was to evaluate some of previously generated HT-producing strains in a 1 L bioreactor to improve the production yield. For this purpose, we used the above-mentioned HT biosynthesis pathway and tried to improve the process by evaluating different production schemes and different induction temperatures. From these experiments, we obtained yields of about 82% of HT production from L-DOPA (DOPA hereafter) and L-tyrosine (tyrosine hereafter) without the need to manipulate the genetic background of the bacteria.

## 2. Materials and Methods

### 2.1. Plasmid and Strain Construction

The construction of the plasmids pRSF:PcAAS:RsTYR, pACYC:PcAAS and pCDF:ALRK is described in [17] (Table 1). Briefly, aromatic acetaldehyde synthase (*PcAAS*) from parsley (*Petroselinum crispum*) and tyrosinase from *Ralstonia solanacearum* were cloned into the expression vector pRSFDuet-1 under the control of distinct T7 promoters to deliver pRSF:PcAAS:RsTYR and pACYC:PcAAS. The cloned *PcAAS* was subcloned to deliver pACYC:PcAAS. Lastly, *E. coli* aldehyde reductase K was cloned into the expression vector pCDFDuet-1 under the control of a T7 promoter to deliver pCDF:ALRK. All constructed plasmids were transformed into BL21(DE3) competent cells. The production of HT from tyrosine was based on the BL21(DE3) strain of *E. coli* harboring the pRSF:PcAAS:RsTYR and pCDF:ALRK plasmids (strain BL^Tyr→HT^) while the production of HT from DOPA was based on the strain harboring pACYC:PcAAS (strain BL^DOPA→HT^). Primer design and in silico DNA manipulations were completed with SnapGene v6.2.2 software and validated with BLAST [26].

### 2.2. Hydroxytyrosol Production in 1 L Bioreactor

For lab-scale HT production, a 1 L bioreactor (BioFlo 120, Eppendorf, Hamburg, Germany) was used. Single colonies from the appropriate glycerol stock were grown for 16 h in LB under selection with the appropriate antibiotic (chloramphenicol for the strain BL^DOPA→HT^, kanamycin and spectinomycin for the strain BL^Tyr→HT^) at 37 °C to generate a preculture. The preculture was centrifuged at 6000× *g* for 10 min at 4 °C, washed and resuspended in minimal M9 salts (22.3 mM Na_2_HPO_4_·7 H_2_O, 22 mM KH_2_PO_4_, 8.6 mM NaCl, 18.7 mM NH_4_Cl, 1 mM MgSO_4_, 25 μM CuSO_4_, 0.1 mM CaCl_2_). This step was repeated twice in order to wash out any remnants of the LB medium. The resuspended bacterial cells were used to inoculate M9 growth medium supplemented with 10 nM thiamine, 55 mM glucose and the appropriate antibiotics to a final OD_600_ of 0.2–0.3. The culture was grown at 37 °C until the OD_600_ reached 0.6–0.8. At that point, Isopropyl β-d-1-thiogalactopyranoside (IPTG) was added to a final concentration of 0.1 mM and the temperature was set to 18 °C or 30 °C (depending on the experiment). Agitation was set to 300 rpm and pH was kept at 6.5, while dissolved oxygen (DO) was constantly kept above 30% upon exogenous supplementation of sterile air at a flow rate of 0.1 standard liters per minute. These three growth parameters were continuously monitored and their changes are depicted in Supplementary Figure S1. After a 20 h incubation period, the temperature was adjusted to 30 °C and the appropriate substrate was added (final concentration of 4 mM DOPA or 2 mM tyrosine). Samples were withdrawn at specific time points for the analysis of tyrosine, DOPA and HT. 

### 2.3. Culture Conditions and Preparation of Whole-Cell Biocatalysts

Single colonies from the appropriate glycerol stock were grown for 16 h in LB with the respective antibiotics at 37 °C to generate the preculture medium. This was used to inoculate 50 mL of LB in a 250 mL Erlenmeyer flask with the appropriate antibiotics to a final OD_600_ of 0.1. The culture was grown at 37 °C until the OD_600_ reached 0.6. At that point, IPTG was added to a final concentration of 0.1 mM and the temperature was set to 18 °C or 30 °C. After a 24 h incubation period, the bacteria were harvested by centrifugation (6000× *g*, 10 min), washed twice with 50 mL ice-cold sterile distilled water and the pellet was weighted and resuspended in 1× M9 minimal salts (*w*/*v*) to deliver a 40 mg/mL cell suspension. The resuspended cells were kept at 4 °C until use. 

### 2.4. Whole-Cell Bioatalysis Reaction

The whole-cell biocatalysis reaction was conducted as described by Li et al. [27]. Briefly, the reaction contained 20 mg/mL cells, 1X minimal M9 salts, 1% (*w*/*o*) filter sterilized glucose and the appropriate substrate in a total volume of 5 mL. Biocatalysis temperature was set to 18 °C or 30 °C. Samples of 100 μL were collected at specific time points and kept at −20 °C until HPLC-MS/MS analysis. For this purpose, the aliquots were diluted in MS grade water.

### 2.5. Liquid Chromatography and Mass Spectrometry Metabolite Analysis

Detection of tyrosine, DOPA and HT was performed using an ultra-high performance liquid chromatography system coupled with a triple quadrupole mass spectrometer (UHPLC-MS/MS, ExionLC, SCIEX QTRAP 4500), a roughing pump and a source of compressed air and nitrogen. Analyte separations were achieved with a reversed phase C18 liquid chromatography column (50 × 2 mm, Synergi Fusion-RP, 4 μm spherical particle size, 80 Å pore size, Phenomenex, Torrance, CA, USA) in an 8 min run time with the column oven constantly kept at 40 °C. A total of 10 μL of each sample was injected at a sampling speed of 5 μL/s. The mobile phase consisted of a mixture of HPLC-MS grade solvents, water (solvent A) and methanol (solvent B), at a flow rate of 0.6 mL/min. Elution was performed with a binary gradient, starting with 98% solvent A and 2% solvent B until 0.6 min, 75% A and 25% B at 2.40 min, 50% A and 50% B at 2.50 min, 30% A and 70% B at 4.80 min, 5% A and 95% B at 4.90 min, 5% A and 95% B at 5.40 min, 98% A and 2% B at 5.50 min and finally 98% A and 2% B at 8.00 min. Tyrosine was eluted at 0.39 min, DOPA at 0.35 min and HT at 0.9 min. All sample vials were maintained at 15 °C during the analysis. After separation, the analytes were diverted to the mass spectrometer module where electrospray ionization (ESI) was performed by a Turbo V ion source in atmospheric pressure. The MS source and gas parameters were as follows: curtain gas (CUR), 35 psi; collision gas (CAD), medium; ion source gas 1 (GS1), 50 psi; ion source gas 2 (GS2), 60 psi; temperature, 600 °C; resolution Q1, unit; resolution Q3, unit; ion-spray voltage (IS), 4500 V and 4500 V in positive and negative ion modes, respectively. The MS/MS conditions, multiple reaction monitoring (MRM) transitions and compound-dependent parameters were identified by infusion. The scan type was MRM with negative ion mode for HT and tyrosine and positive ion mode for DOPA. The MRM transitions used for qualitative and quantitative detection and the compound-dependent parameters are presented in Table 2. Infusion, transition scanning and data acquisition were performed using Analyst software (SCIEX, version 1.7.1) and data analysis was performed using SCIEX OS software (version 1.7.0.36606). A seven-point linear calibration curve was generated for each compound over a range of 5–500 ng/mL (r^2^ > 0.99) in SCIEX OS and the regression weighting type was 1/x. Accuracy ranged from 92 to 99% for all the compounds analyzed.

## 3. Results

### 3.1. Scaling up Flask Experiments to Produce Hydroxytyrosol from DOPA in a Bioreactor

**Figure 1:**
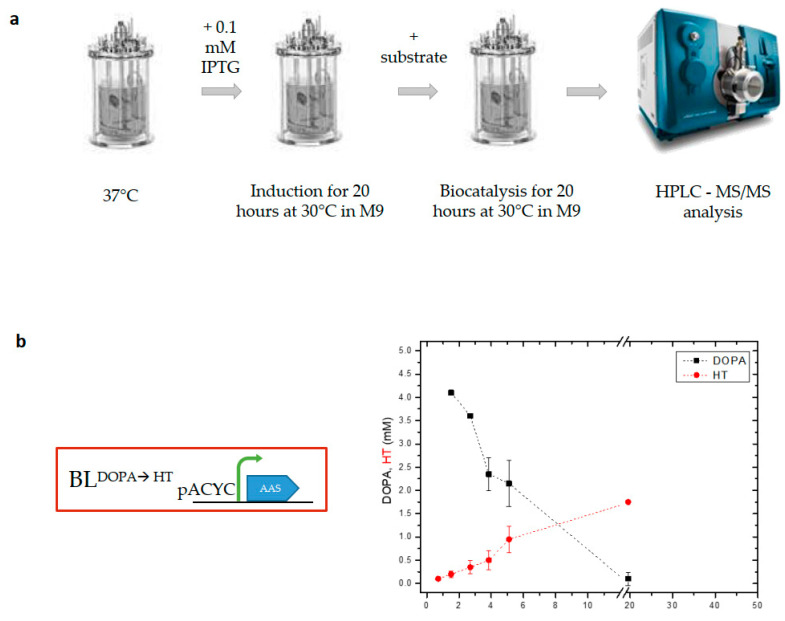
Whole-cell biocatalysis experiments in a 1 L bioreactor. (**a**) Schematic representation of the experimental procedure used. Induction of heterologously expressed proteins in strain BL^DOPA→HT^ and biocatalysis were performed at 30 °C. (**b**) Diagram showing the bioconversion of DOPA into hydroxytyrosol (HT). An average HT yield of 45.5% ± 12 was reached as estimated by the initial DOPA concentration added. Three biological replicates were used for the experiments.

To evaluate the production of HT from DOPA in a 1 L bioreactor, we used the BL21(DE3) *E. coli* strain expressing aromatic acetaldehyde synthase from *Petroselinum crispum* (PcAAS) (strain BL^DOPA→HT^, Table 1). PcAAS catalyzes the concurrent decarboxylation and deamination of DOPA, leading to the production of 3,4-dihydroxyphenyl acetaldehyde (DHPAA), which is subsequently converted to HT via an endogenously expressed aldehyde reductase (ALRK). The experimental procedure in the bioreactor followed the protocol used in the flask experiments [17]. Briefly, when the OD_600_ of a fresh culture reached 0.6 at 37 °C, IPTG was added (0.1 mM) and the temperature was reduced to 30 °C. Twenty hours after the induction, DOPA was added to a final concentration of 4 mM. During the bioreaction process, aliquots of the culture were collected and used to measure various parameters (OD_600_ concentrations of DOPA and HT) (Figure 1) while growth parameters (pH, temperature, DO) were monitored during the whole process and kept at fixed ranges. Five hours after the addition of the substrate, 50% of it was metabolized, while at the end of the experiment (20 h after the addition of the substrate), the concentration of DOPA was under the detection levels of HPLC-MS/MS. Despite the absence of DOPA in the medium, the production of HT did not increase proportionally. At the end of the biocatalysis period, DOPA was converted into HT, reaching a relatively low yield of 45.5% ± 12 (Figure 1b). 

### 3.2. Evaluation of Different Induction Temperatures Utilizing a Whole-Cell Biocatalysis Approach

In flask experiments, the production of biocatalytically active cells and the production phase are conducted in the same temperature and growth medium. This is not always the optimal condition, since *E. coli* growth at commonly used temperatures (30–37 °C) usually leads to increased protein expression levels, which in turn might lead to the misfolding of heterologous proteins and the formation of inclusion bodies [28]. In such cases, lowering the expression level, leading to a smaller amount of heterologously expressed proteins, might be beneficial. 

**Figure 2:**
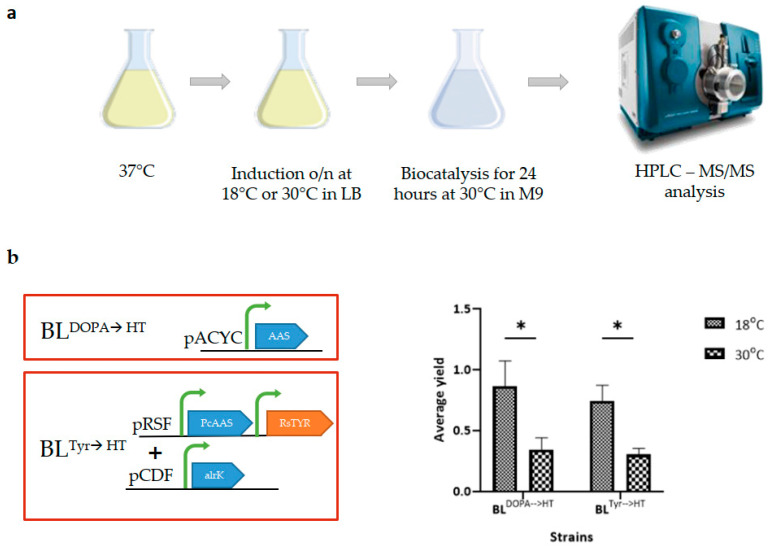
Whole-cell biocatalysis experiments for the production of hydroxytyrosol (HT) from various substrates. (**a**) Schematic presentation of the experimental procedure used. Two different temperatures, 18 °C and 30 °C, were used for the induction of the heterologously expressed protein in each strain. Two different strains of *E. coli* were used for the production of HT, one from tyrosine (strain BL^Tyr→HT^) and one from DOPA (strain BL^DOPA→HT^). (**b**) Diagram showing the average yield of HT production from various substrates at the two different temperatures used. In all cases, higher yields of HT were observed when induction was performed at 18 °C. Statistical analysis was performed with one-way ANOVA (* *p* < 0,05; 2–3 biological replicates were used for each experiment).

Since this might also have been the case in our experiments, we tested the whole-cell biocatalysis method with HT-producing strains in a small-scale set-up. In these experiments, in addition to the BL^DOPA→HT^ strain, the BL^Tyr→HT^ strain was included. This strain of *E. coli* heterologously expresses tyrosinase from *Rasltonia solanacerarum* (*Rs*TYR) and *Pc*AAS and overexpresses a native ALRK. Thus, the substrate, tyrosine, is metabolized to HT by the diphenol or monophenol route [17]. In this experiment (Figure 2a), expression of heterologous proteins was induced in LB flasks previously inoculated with the proper strain (strain BL^Tyr→HT^ or BL^DOPA→HT^). For HT bioconversion, cell pellets of the overnight-grown cultures were incubated in M9 in the presence of the appropriate substrate (tyrosine or DOPA, respectively). 

To study whether temperature was a rate-limiting factor for HT production, we induced gene expression at two temperatures: 18 °C and 30 °C. The resulting biocatalysts (whole-cell pellets) were evaluated for their ability to produce HT through biocatalysis performed at the same temperature as in the flask experiments (30 °C). Supernatants collected at 24 h post substrate addition were analyzed by HPLC-MS/MS. As shown in Figure 2b, all biocatalysts induced at 18 °C outperformed those induced at 30 °C, reaching 90% ± 20.6 and 74% ± 13.3 HT yields in the BL^DOPA→HT^ and BL^Tyr→HT^ strains, respectively, in 24 h.

### 3.3. Implementation of a Modified Whole-Cell Biocatalysis Method in a 1 L Bioreactor Led to Increased HT Yields

**Figure 3:**
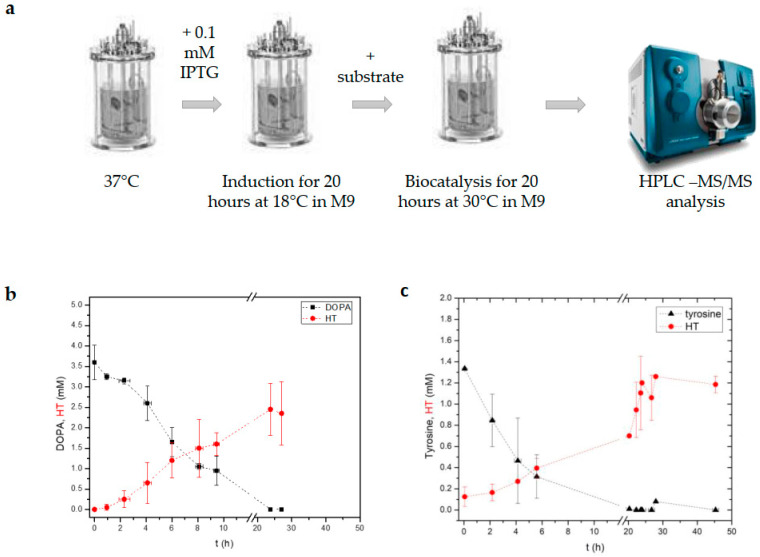
Whole-cell biocatalysis experiments in a 1 L bioreactor. (**a**) Schematic representation of the experimental procedure used. Induction of heterogously expressed proteins in each strain was performed at 18 °C while biocatalysis was performed at 30 °C. Two different strains of *E. coli* were used for the production of hydroxytyrosol (HT), one from tyrosine (strain B^Tyr→HT^) and one from DOPA (strain B^DOPA→HT^). (**b**) Diagram showing the production of HT vs. the substrate for strain BL^DOPA→HT^ and strain BL^Tyr→HT^. (**c**) The average yield for strain BL^DOPA→HT^ (two independent experiments) was 82% ± 9 (two independent experiments), for strain BL^Tyr→HT^, it was 81% ± 14 (two independent experiments) as estimated by the initial precursor concentration added.

Since the whole-cell biocatalysis method led to elevated HT production yields, we sought to implement similar conditions in the 1 L bioreactor. To simplify the procedure in the bioreactor, we used a modified biocatalysis protocol (Figure 3a). Instead of using the LB medium for the induction process and the M9 medium for the subsequent reaction, we induced the culture in the M9 medium at 18 °C for 20 h and elevated the temperature to 30 °C just before adding the substrate. During the bioreaction, aliquots of the culture were collected and used for metabolite analysis (Figure 3b,c). As shown in Figure 3b, DOPA (4 mM) could be effectively metabolized into HT, reaching yields as high as 81% ± 14. During this process, almost 50% of DOPA had been metabolized in the first five hours of biocatalysis, while at the end of the experiment (20 h after substrate addition), the concentration of DOPA was at undetectable limits. Next, we sought to evaluate the same method using the BL^Tyr→HT^ strain and performing, essentially, the same process. Again, we observed an 82% ± 9 HT yield from the initial substrate tyrosine (Figure 3c). As in the case of the BL^DOPA→HT^ strain, in the first six hours, 80% of tyrosine had been metabolized, while HT reached its maximum production 16 h later. Altogether, by lowering the induction temperature, we were able to increase HT yields. 

## 4. Discussion

The aim of this study was to evaluate and improve the ability of two HT-producing *E. coli* strains, previously constructed and evaluated in shake flask fed-batch experiments [17] in a 1 L bioreactor. Initially, we used the strain BL^DOPA→HT^, which heterologously expresses one gene (*PcAAS*) for the production of HT from DOPA. For this purpose, we implemented the same conditions we had used in flask experiments in the 1 L bioreactor. Despite this, we found a mere 45% yield of HT when using DOPA as a substrate. In order to increase the yield, we used a small-scale whole-cell biocatalysis protocol to investigate the role of temperature in HT production. In these experiments, we included a second strain we had engineered [17], the strain BL^Tyr→HT^, which is able to bioconvert tyrosine into HT utilizing a dual pathway approach due to the double specificity of the expressed genes. By comparing induction temperatures (18 °C vs. 30 °C), we succeeded in reaching higher HT yields (90% and 74% from DOPA and tyrosine, respectively). Returning to the bioreactor, we applied different temperatures during the induction (18 °C) and biocatalysis (30 °C) steps and succeeded in increasing HT production yields in both strains, reaching an 81% and 82% yield in strains BL^DOPA→HT^ and BL^Tyr→HT^, respectively. 

In bioreactors, contrary to shake flask fed-batch experiments, the parameters affecting bacterial growth, like pH and DO, are largely controlled. This leads to optimal growth conditions for bacterial strains, resulting in higher expression levels of heterologously expressed genes. But the increased protein yield does not necessarily lead to protein functionality. As is widely documented, protein misfolding is a major concern in the production of recombinant proteins (reviewed by [28] ). One of the easiest strategies that have been employed to circumvent such issues relates to the modification of growth conditions, and more specifically of induction temperature [28].

To test whether a decrease in induction temperature might have a positive effect on HT production, we applied the whole-cell biocatalysis method on a small scale to test the effect of two different temperatures on HT production. We preferred this process over routinely used fed-batch experiments because we aimed to decouple the induction and the biocatalysis phases. From the outcome of our experiments, it can be concluded that by applying a lower induction temperature (18 °C vs. 30 °C), higher HT yields were obtained. This positive effect on HT production was observed for both of the strains used in this study (BL^DOPA→HT^ and B^Tyr→HT^). It has been widely documented that temperature affects all biological systems either directly (through protein folding, stability, enzyme activity, etc.) or indirectly through changes in gene expression or membrane fluidity (reviewed in [29]). At this point, we cannot designate a specific reason for the increase in HT yield due to the lower induction temperature used. The higher expression levels achieved at higher induction temperatures probably led to the formation of aggregates in inclusion bodies (IB), a phenomenon commonly reported in the use of strong promoters (as in our case) in heterologous protein expression systems, as described in [30].

To date, different metabolic pathways have been used in order to produce HT in various microorganisms [14], mainly on a small scale, but only a few of them have been validated or implemented in larger-volume bioreactors [22,23,24,25]. Thus, from these few studies, one cannot conclude on the possible bottlenecks in HT production at higher volumes. According to our study and the study of Brouk et al. [23], decoupling the growth and bioconversion phases seems to be a crucial factor in increasing yield. Another factor leading to elevated yields of HT in bioreactors can be obtained by generating more versatile strains. This was the case in previous reports. More specifically, it was sequential genetic manipulation of the original strains used in fed-batch experiments that led to strain improvement and subsequently to higher HT yields [22,23]. This is a laborious process, in terms of gene manipulation but also in practical terms, since in these studies, large volumes of bacterial cultures grown in a bioreactor must first be processed and then used for biocatalysis. In this study, we improved the HT yield of metabolically engineered strains without the requirement of any additional improvement in the genetic background of the strains used or the necessity to change the medium of the bacterial culture before the biocatalysis procedure.

## Figures and Tables

**Table 1:** Metabolically engineered bacterial plasmids and strains used in this work.

Plasmid Name	Genotype
pRSFDuet-1	RSF ori kan lacI T7prom T7term
pCDFDuet-1	CDF ori aadA lacI T7prom T7term
pACYCDuet-1	P15A ori CmR lacI T7prom T7term
pRSF:TYR	RSF ori kan lacI T7prom:RsTYR:T7term [17]
pACYC:AAS	P15A ori CmR lacI T7prom:PcAAS:T7term [17]
pCDF:ALRK	CDF ori aadA lacI T7prom:EcALRK:T7term [17]
**Strain name**	**Genotype**
BL21(DE3)	F-ompT gal dcm lon hsdSB(rB - mB -) λ(DE3 [lacI lac-UV5-T7 gene 1 ind1 sam7 nin5])
BL^Tyr→HT^	BL21(DE3) pRSF:TYR:AAS pCDF:ALRK [17]
BL^DOPA→HT^	BL21(DE3) pACYC:AAS [17]

**Table 2:** MRM transitions monitored on the SCIEX QTRAP 4500 MS-MS module (Q1, Q3 masses) and acquisition method conditions for each compound (dwell time; declustering potential, DP; entrance potential, EP; collision energy, CE; collision cell exit potential, CXP).

Analyte	Q1 Mass (Da)	Q3 Mass (Da)	Dwell Time (msec)	DP (V)	EP (V)	CE (V)	CXP (V)
Hydroxytyrosol	153.00	123.00	50.0	−25.00	−10.00	−22.00	−9.00
Tyrosine	180.00	162.80	50.0	−75.00	−10.00	−18.00	−7.00
DOPA	198.00	152.00	50.0	70.00	10.00	19.00	9.00

## Data Availability

All data are available on request. The data are not publicly available due to privacy.

## Supplementary Materials

The following supporting information can be downloaded at: https://www.mdpi.com/article/10.3390/metabo13111126/s1, Figure S1: Monitoring of the main growth parameters.

## References

[1] Karković Marković A., Torić J., Barbarić M., Jakobušić Brala C. (2019). Hydroxytyrosol, Tyrosol and Derivatives and Their Potential Effects on Human Health. Molecules.

[2] Nikou T., Sakavitsi M.E., Kalampokis E., Halabalaki M. (2022). Metabolism and Bioavailability of Olive Bioactive Constituents Based on In Vitro, In Vivo and Human Studies. Nutrients.

[3] Zoidou E., Melliou E., Gikas E., Tsarbopoulos A., Magiatis P., Skaltsounis A.-L. (2010). Identification of Throuba Thassos, a Traditional Greek Table Olive Variety, as a Nutritional Rich Source of Oleuropein. J. Agric. Food Chem..

[4] Bendini A., Cerretani L., Carrasco-Pancorbo A., Gómez-Caravaca A., Segura-Carretero A., Fernández-Gutiérrez A., Lercker G. (2007). Phenolic Molecules in Virgin Olive Oils: A Survey of Their Sensory Properties, Health Effects, Antioxidant Activity and Analytical Methods. An Overview of the Last Decade Alessandra. Molecules.

[5] Agalias A., Magiatis P., Skaltsounis A.-L., Mikros E., Tsarbopoulos A., Gikas E., Spanos I., Manios T. (2007). A New Process for the Management of Olive Oil Mill Waste Water and Recovery of Natural Antioxidants. J. Agric. Food Chem..

[6] Granados-Principal S., Quiles J.L., Ramirez-Tortosa C.L., Sanchez-Rovira P., Ramirez-Tortosa M.C. (2010). Hydroxytyrosol: From Laboratory Investigations to Future Clinical Trials. Nutr. Rev..

[7] Robles-Almazan M., Pulido-Moran M., Moreno-Fernandez J., Ramirez-Tortosa C., Rodriguez-Garcia C., Quiles J.L., Ramirez-Tortosa M. (2018). Hydroxytyrosol: Bioavailability, Toxicity, and Clinical Applications. Food Res. Int..

[8] EFSA Panel on Dietetic Products, Nutrition and Allergies (NDA) (2011). Scientific Opinion on the Substantiation of Health Claims Related to Polyphenols in Olive and Protection of LDL Particles from Oxidative Damage (ID 1333, 1638, 1639, 1696, 2865), Maintenance of Normal Blood HDL-Cholesterol Concentrations (ID 1639), Maintenance of Normal Blood Pressure (ID 3781), “Anti-Inflammatory Properties” (ID 1882), “Contributes to the Upper Respiratory Tract Health” (ID 3468), “Can Help to Maintain a Normal Function of Gastrointestinal Tract” (3779), and “Contributes to Body Defences against External Agents” (ID 3467) Pursuant to Article 13(1) of Regulation (EC) No 1924/2006. EFSA J..

[9] Kyriazis J.D., Aligiannis N., Polychronopoulos P., Skaltsounis A.-L., Dotsika E. (2013). Leishmanicidal Activity Assessment of Olive Tree Extracts. Phytomedicine.

[10] Crisante F., Taresco V., Donelli G., Vuotto C., Martinelli A., D’Ilario L., Pietrelli L., Francolini I., Piozzi A., Donelli G. (2015). Antioxidant Hydroxytyrosol-Based Polyacrylate with Antimicrobial and Antiadhesive Activity Versus Staphylococcus Epidermidis. Advances in Microbiology, Infectious Diseases and Public Health.

[11] Bisignano G., Tomaino A., Cascio R.L., Crisafi G., Uccella N., Saija A. (2010). On the In-Vitro Antimicrobial Activity of Oleuropein and Hydroxytyrosol. J. Pharm. Pharmacol..

[12] Medina-Martínez M.S., Truchado P., Castro-Ibáñez I., Allende A. (2016). Antimicrobial Activity of Hydroxytyrosol: A Current Controversy. Biosci. Biotechnol. Biochem..

[13] Sun X., Li X., Shen X., Wang J., Yuan Q. (2021). Recent Advances in Microbial Production of Phenolic Compounds. Chin. J. Chem. Eng..

[14] Britton J., Davis R., O’Connor K.E. (2019). Chemical, Physical and Biotechnological Approaches to the Production of the Potent Antioxidant Hydroxytyrosol. Appl. Microbiol. Biotechnol..

[15] Chung D., Kim S.Y., Ahn J.-H. (2017). Production of Three Phenylethanoids, Tyrosol, Hydroxytyrosol, and Salidroside, Using Plant Genes Expressing in *Escherichia coli*. Sci. Rep..

[16] Choo H.J., Kim E.J., Kim S.Y., Lee Y., Kim B.-G., Ahn J.-H. (2018). Microbial Synthesis of Hydroxytyrosol and Hydroxysalidroside. Appl. Biol. Chem..

[17] Trantas E., Navakoudis E., Pavlidis T., Nikou T., Halabalaki M., Skaltsounis L., Ververidis F. (2019). Dual Pathway for Metabolic Engineering of *Escherichia coli* to Produce the Highly Valuable Hydroxytyrosol. PLoS ONE.

[18] Li X., Chen Z., Wu Y., Yan Y., Sun X., Yuan Q. (2018). Establishing an Artificial Pathway for Efficient Biosynthesis of Hydroxytyrosol. ACS Synth. Biol..

[19] Chen W., Yao J., Meng J., Han W., Tao Y., Chen Y., Guo Y., Shi G., He Y., Jin J.-M. (2019). Promiscuous Enzymatic Activity-Aided Multiple-Pathway Network Design for Metabolic Flux Rearrangement in Hydroxytyrosol Biosynthesis. Nat. Commun..

[20] Liebgott P.-P., Amouric A., Comte A., Tholozan J.-L., Lorquin J. (2009). Hydroxytyrosol from Tyrosol Using Hydroxyphenylacetic Acid-Induced Bacterial Cultures and Evidence of the Role of 4-HPA 3-Hydroxylase. Res. Microbiol..

[21] Allouche N., Sayadi S. (2005). Synthesis of Hydroxytyrosol, 2-Hydroxyphenylacetic Acid, and 3-Hydroxyphenylacetic Acid by Differential Conversion of Tyrosol Isomers Using *Serratia marcescens* Strain. J. Agric. Food Chem..

[22] Bouallagui Z., Sayadi S. (2018). Bioconversion of p -Tyrosol into Hydroxytyrosol under Bench-Scale Fermentation. BioMed Res. Int..

[23] Brouk M., Fishman A. (2012). Improving Process Conditions of Hydroxytyrosol Synthesis by Toluene-4-Monooxygenase. J. Mol. Catal. B Enzym..

[24] Bernath-Levin K., Shainsky J., Sigawi L., Fishman A. (2014). Directed Evolution of Nitrobenzene Dioxygenase for the Synthesis of the Antioxidant Hydroxytyrosol. Appl. Microbiol. Biotechnol..

[25] Koma D., Fujisawa M., Ohashi H., Yamanaka H., Moriyoshi K., Nagamori E., Ohmoto T. (2023). Production of 3-Hydroxytyrosol from Glucose by Chromosomally Engineered *Escherichia coli* by Fed-Batch Cultivation in a Jar Fermenter. J. Agric. Food Chem..

[26] Altschul S.F., Gish W., Miller W., Myers E.W., Lipman D.J. (1990). Basic Local Alignment Search Tool. J. Mol. Biol..

[27] Li C., Jia P., Bai Y., Fan T., Zheng X., Cai Y. (2019). Efficient Synthesis of Hydroxytyrosol from l -3,4-Dihydroxyphenylalanine Using Engineered *Escherichia coli* Whole Cells. J. Agric. Food Chem..

[28] Bhatwa A., Wang W., Hassan Y.I., Abraham N., Li X.Z., Zhou T. (2021). Challenges Associated With the Formation of Recombinant Protein Inclusion Bodies in *Escherichia coli* and Strategies to Address Them for Industrial Applications. Front. Bioeng. Biotechnol..

[29] Knapp B.D., Huang K.C. (2022). The Effects of Temperature on Cellular Physiology. Annu. Rev. Biophys..

[30] Fahnert B., Lilie H., Neubauer P. (2004). Inclusion Bodies: Formation and Utilisation. Physiological Stress Responses in Bioprocesses.

