# Supplementary material for: An Engineered Plant Metabolic Pathway Results in High Yields of Hydroxytyrosol Due to a Modified Whole-Cell Biocatalysis in Bioreactor
Source: Metabolites. 2023 Nov 3;13(11):1126. doi: 10.3390/metabo13111126 (PMC10672836; doi:10.3390/metabo13111126)
Supplement: Supplementary file 1 [file metabolites-13-01126-s001.zip › metabolites-2674471-supplementary.pdf]

Supplemented Material

An engineered plant metabolic pathway results in high yields of Hydroxytyrosol due to a modified whole-cell biocatalysis in bioreactor

Glykeria Mermigka[1,2,#], Aikaterini I. Vavouraki[1,#], Chrysoula Nikolaou[1], Ioanna Cheiladaki[1], Michail Vourexakis[1], Dimitrios Goumas[1,2], Filippos Ververidis[1,2,*] and Emmanouil Trantas[1,2,*]

[1] Hellenic Mediterranean University (HMU), School of Agricultural Sciences, Department of Agriculture, Laboratory of Biological and Biotechnological Applications (LBBA), GR71004, Heraklion, Greece; GM, gmermigka@hmu.gr; AV, avavouraki@hmu.gr; CN, xrica.nikolaou@gmail.com; IC, icheiladaki@hmu.gr; MV, michaelvourexakis@yahoo.com; DG, dgoumas@hmu.gr; FV, ververidis@hmu.gr; ET, mtrantas@hmu.gr

[2] Hellenic Mediterranean University, University Research Centre, Institute of Agri-Food and Life Sciences, GR71410, Heraklion, Greece

[#] These authors contributed equally

***** Correspondence: ET, mtrantas@hmu.gr; FV, ververidis@hmu.gr

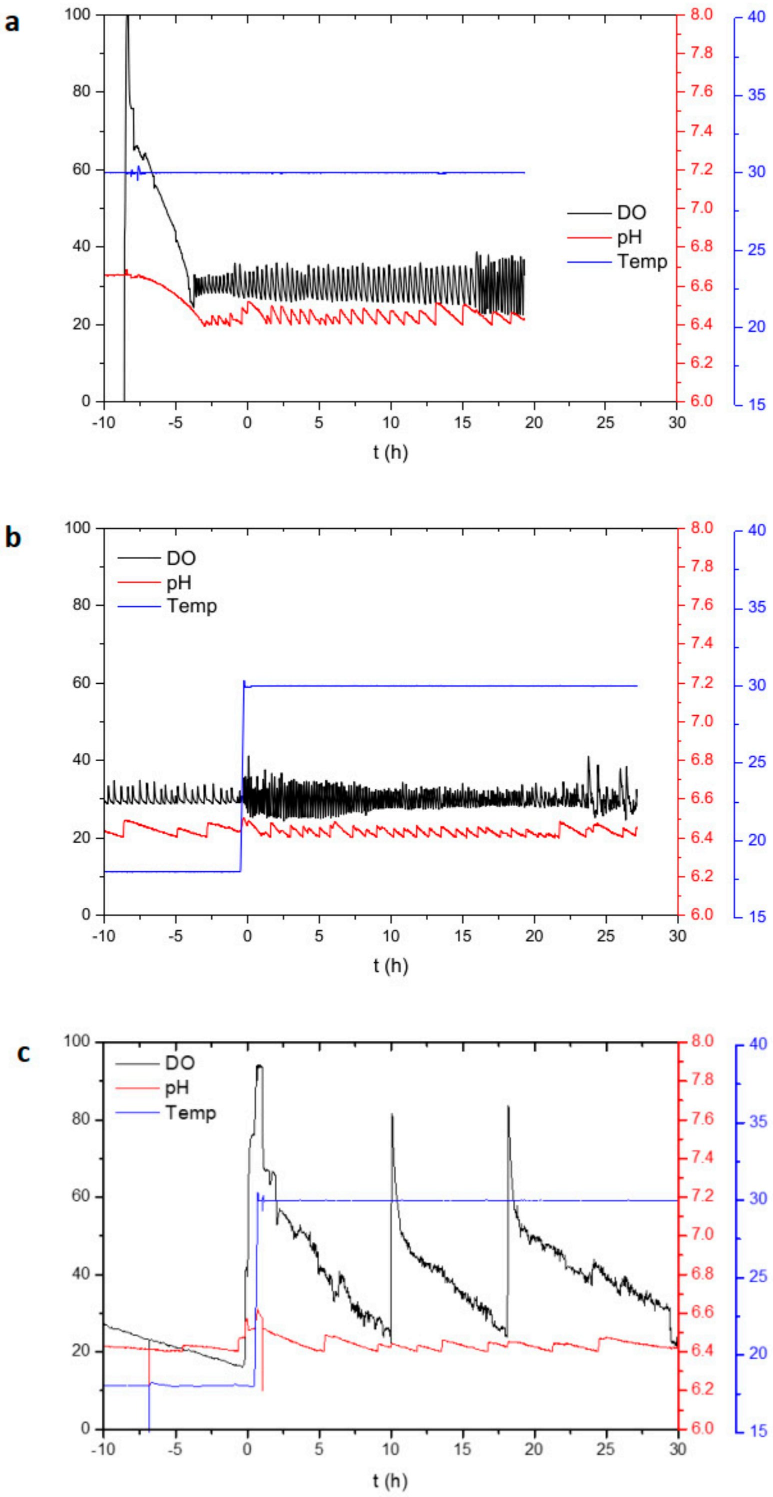

Figure S1. The changes of the growth parameters, dissolved oxygen (DO), temperature (Temp), and pH, were recorded during the whole experiment and plotted into charts (a, b, c). **a** and **b:** plots corresponding to the growth parameters for strain BL$^{DOPA \rightarrow HT}$ (30°C induction temperature and 18°C induction temperature, respectively) and **c:** plot corresponding to the growth parameters for strain BL$^{Tyr \rightarrow HT}$.
